# Comparison of Efficacy and Safety of Denosumab with Eldecalcitol or Native Vitamin D in Postmenopausal Chinese Women with Osteoporosis (ESCORT): A Randomized Controlled Trial

**DOI:** 10.3390/jcm15145570

**Published:** 2026-07-16

**Authors:** Yuhong Zeng, Qinghua Tang, Jiancheng Yang, Qingmei Li, Lei Yang, Bin Zhang, Ming Yang, Maohong Che, Yuhan Peng

**Affiliations:** Department of Osteoporosis, Honghui Hospital, Xi’an Jiaotong University, Xi’an 710054, China; gshua691994@163.com (Q.T.); yjchhyy2021@163.com (J.Y.); liqingmei09@163.com (Q.L.); yleii82@126.com (L.Y.); zb7744@163.com (B.Z.); 18591776708@163.com (M.Y.); chemhh408@163.com (M.C.); p1846507987@gmail.com (Y.P.)

**Keywords:** postmenopausal osteoporosis, bone mineral density, bone turnover markers, denosumab, eldecalcitol

## Abstract

**Background:** Eldecalcitol (ELD) and denosumab are some of the most common therapeutic options for osteoporosis management. ELD effectively increases bone mineral density (BMD) in osteoporotic patients, independent of baseline vitamin D status or calcium intake. However, the efficacy of denosumab combined with either ELD or native vitamin D plus calcium in postmenopausal Chinese women with osteoporosis has not been established. **Methods:** In this single-center, randomized, open-label, active-controlled clinical trial, postmenopausal women with osteoporosis (defined as a BMD T-score ≤ −2.5 at the lumbar spine [LS], total hip [TH], or femoral neck [FN], or low bone mass with fragility fracture history) were enrolled from Xi’an Honghui Hospital in China and randomized 1:1 to two 12-month treatment regimens: the ELD group received ELD (0.75 μg orally daily) combined with denosumab (60 mg subcutaneously every 6 months), while the control group received the same denosumab regimen plus native vitamin D (800 IU orally daily) and calcium (600 mg orally daily). The primary endpoint was the 12-month percent change in LS BMD from baseline. Secondary endpoints included changes in FN- and TH-BMD from baseline, bone turnover markers, serum parathyroid hormone, quality of life, and the incidence of new fractures. **Results:** Of the 100 randomized participants, 45 in the ELD group and 46 in the control group were included in the efficacy analysis. After 12 months of treatment, LS-BMD increased significantly in both groups (both *p* < 0.05), with a greater increase in the ELD group than in the control group (6.75% vs. 4.99%), yielding a statistically significant between-group least-squares mean difference of 1.75% (95% CI, 0.10 to 3.41; *p* = 0.038). The reduction in serum β-CTX was significantly smaller in the ELD group than in the control group at 3 months (91.00% vs. 93.62%, *p* = 0.002), with no significant between-group differences thereafter. Seven fractures were reported (one non-vertebral in the ELD group; one vertebral and five non-vertebral in the control group). No significant between-group differences in FN- or TH-BMD, quality of life, or overall adverse event rates were observed. Both regimens were generally well-tolerated, without clinically meaningful calcium-related safety signals. **Conclusions:** Combination therapy with denosumab and eldecalcitol improved LS-BMD more effectively than denosumab with native vitamin D and calcium in postmenopausal Chinese women with osteoporosis. Clinical trial number: ClinicalTrials.gov identifier NCT05884372, registered on 1 June 2023.

## 1. Introduction

Osteoporosis is a metabolic bone disease characterized by decreased bone mass and increased fracture risk [1,2]. It affects more than 200 million women worldwide, with postmenopausal women being disproportionately susceptible to the disease and its severe sequelae, particularly osteoporotic fractures [3,4]. In China, the prevalence of osteoporosis exceeded 29% among women aged over 50 [5], with approximately 40% of these patients expected to experience fragility fractures; it is therefore a growing concern in the modern aging society [6].

Denosumab is a monoclonal antibody that inhibits osteoclastic bone resorption by binding to receptor activator of nuclear factor-κB ligand (RANKL), thereby suppressing osteoclast development, function, and survival [7]. Denosumab has been shown to significantly reduce the risk of vertebral, hip, and non-vertebral fractures across multiple clinical settings [8,9,10]. Despite its well-established antiresorptive efficacy, denosumab therapy is occasionally associated with hypocalcemia, necessitating concomitant supplementation with calcium and vitamin D to maintain calcium homeostasis [11]. In clinical practice, both native vitamin D and active vitamin D analogs such as eldecalcitol (ELD) are commonly co-administered with denosumab to prevent this complication and optimize skeletal benefits [12].

ELD is an active vitamin D3 [2β-(3-hydroxypropyloxy)-1,25-dihydroxyvitamin D_3_] analog and a vitamin D receptor agonist developed for osteoporosis treatment. Unlike other active vitamin D analogs such as alfacalcidol, it contains a hydroxypropyloxy substituent at the 2β position, conferring greater metabolic stability and selective skeletal effects [13]. ELD also has higher binding affinity for vitamin D-binding protein and a longer half-life than calcitriol, which may contribute to its sustained biological activity and once-daily dosing [13]. It has been approved for treating osteoporosis in Japan and, in 2020, for postmenopausal osteoporosis in China. ELD increases bone mineral density (BMD), reduces the incidence of new vertebral and wrist fractures [14], and enhances intestinal calcium absorption in osteoporotic patients [15]. Previous clinical studies have suggested that the type of vitamin D used in combination with denosumab may influence treatment outcomes; for example, in a Japanese study, denosumab combined with alfacalcidol resulted in greater increases in femoral neck (FN) and distal forearm BMD than denosumab combined with native vitamin D, without significant differences in bone turnover markers between regimens [16]. A subsequent study showed that denosumab combined with ELD produced greater gains in FN-BMD than denosumab combined with native vitamin D in Japanese postmenopausal women with osteoporosis [17].

To date, evidence comparing denosumab combined with active vitamin D analog (ELD) versus native vitamin D plus calcium in Chinese postmenopausal women with osteoporosis remains unavailable. To address this gap, we conducted a single-center, randomized, open-label, active-controlled trial to compare the efficacy and safety of these two denosumab-based regimens.

## 2. Materials and Methods

### 2.1. Study Design and Interventions

This was a single-center, randomized, open-label, active-controlled trial designed to compare the efficacy and safety of denosumab plus ELD (ELD group) versus denosumab plus native vitamin D and calcium (control group) in postmenopausal women with osteoporosis. Eligible participants were randomly assigned to groups in a 1:1 ratio to receive denosumab (60 mg subcutaneously every 6 months; Prolia^®^, Amgen, Thousand Oaks, CA, USA) combined with either ELD (0.75 μg orally daily; EDIROL^®^, Chugai Pharmaceutical China Co., Ltd., Shanghai, China) or native vitamin D (800 IU orally daily; Yikexin®, Shandong Dyne Marine Biopharmaceutical Co., Ltd., Rongcheng, China) plus calcium (600 mg orally daily; Caltrate^®^, Haleon [Suzhou] Pharmaceutical Co., Ltd., Suzhou, China) for 12 months. Calcium supplementation was protocol-mandated in the control group only and not in the ELD group, reflecting our comparison of two adjunctive clinical strategies during denosumab therapy. Dietary calcium intake was not prospectively quantified in this trial.

The randomization schedule was stratified by serum 25(OH)D level (<20 ng/mL and ≥20 ng/mL) at baseline. Eligible participants were then randomized in a 1:1 ratio using a block randomization scheme. Allocation concealment was ensured using sealed envelopes containing treatment codes corresponding to sequential randomization numbers. Once a participant was confirmed eligible for randomization, the next sequential randomization number was assigned, and the corresponding coded envelope determined treatment allocation.

This study was conducted with approval from the Institutional Review Board of Honghui Hospital, Xi’an Jiaotong University (Approval No. 2023-IRB-001, 13 March 2023), in accordance with the Declaration of Helsinki and International Council for Harmonization Good Clinical Practice guidelines. Written informed consent was obtained from all participants before any study procedures were performed. This study was registered on ClinicalTrials.gov with the identifier NCT05884372 on 1 June 2023.

### 2.2. Participants

Postmenopausal women with osteoporosis who voluntarily provided written informed consent were enrolled. All participants were Chinese women of Han ethnicity. Postmenopausal status was defined as spontaneous cessation of menstruation for at least 12 consecutive months, bilateral oophorectomy, or age ≥ 60 years with a documented history of menopause. Osteoporosis was diagnosed according to the following criteria: a BMD T-score ≤ −2.5 at the lumbar spine (LS), total hip (TH), FN, or distal one-third of the radius, as measured by dual-energy X-ray absorptiometry (DXA); or a history of fragility fracture at the vertebrae or hip; or low mineral density (−2.5 < T-score < −1.0) at any of these sites with a fragility fracture of the proximal humerus, pelvis, or distal forearm.

Participants were excluded if they had conditions associated with secondary osteoporosis, including hyperthyroidism, Cushing’s syndrome, hypogonadism, or poorly controlled diabetes mellitus (glycated hemoglobin [HbA1c > 9.0%]). Participants were also excluded if they had received denosumab at any point prior, oral bisphosphonates for more than 3 years or within the 6-month period prior to screening, or other bone-active treatments (glucocorticoids, vitamin K, active vitamin D compounds, selective estrogen receptor modulators, calcitonin, teriparatide, or hormone replacement therapy) within 8 weeks prior to study enrollment. Other exclusion criteria included urinary tract stones detected using B-mode ultrasonography or a prior history of urolithiasis at screening; abnormal calcium metabolism, including corrected serum calcium >2.6 mmol/L or <2.12 mmol/L; hypercalciuria, defined as urinary calcium excretion corrected for glomerular filtrate (GF) > 0.4 mg/dL GF (0.1 mmol/L GF); chronic kidney disease (eGFR < 30 mL/min/1.73 m^2^); a current malignancy or a history of malignancy; or being deemed unsuitable for participation by the investigators.

### 2.3. Endpoints

The primary endpoint was the percent change from baseline in LS-BMD at 12 months between postmenopausal women with osteoporosis treated with denosumab plus ELD and those treated with denosumab plus native vitamin D and calcium. Secondary endpoints included changes in BMD at the LS (L1–L4), femoral neck, and total hip; bone turnover markers, including serum β-isomerized C-terminal telopeptide of type I collagen (β-CTX) and procollagen type I N-terminal propeptide (PINP); serum parathyroid hormone (PTH); quality of life at 6 and 12 months; and the incidence of new vertebral and non-vertebral fractures within 12 months.

Safety was assessed by summarizing adverse events (AEs), serious AEs and changes in safety laboratory analytes (hematology, serum biochemistry, urinalysis, calcium-related tests, 25(OH)D, and PTH) occurring within 12 months in postmenopausal women with osteoporosis.

### 2.4. Measurements

The BMD of the LS (L1–L4), femoral neck, and total hip was measured using a Hologic Discovery A DXA scanner (Hologic Inc., Bedford, MA, USA). For each participant, the same DXA instrument was used throughout the study, and all scans were performed by the same physician using the same scan mode. At least two evaluable vertebrae within L1–L4 were required for analysis. The left total hip was scanned preferentially unless unavailable, in which case the right hip was scanned; the same hip side was maintained for follow-up scans. Scans with marked scoliosis, sclerosis, trauma, or degenerative joint disease precluding reliable analysis were excluded. For follow-up assessments, the previous scan analysis was recalled and the copy/match function was used to ensure consistent size, shape, and position of the region of interest across visits. In this open-label trial, DXA scans were analyzed in a blinded manner, as the physician responsible for DXA analysis was unaware of the treatment allocation.

Longitudinal quality control (QC) was performed using repeated phantom scans according to the study protocol, and participant scans were performed only when phantom measurements were within the predefined acceptable range. Briefly, before the first participant was enrolled, the spine phantom at each center was scanned 10 consecutive times without repositioning or changing scan settings to establish the mean baseline BMD and an acceptable longitudinal QC range of ±1.5%. The same QC phantom, scan mode, and scan region were used throughout the study. Phantom scans were performed before participant scanning on each study day; if no participant was scheduled in a given week, phantom scanning was performed at least three times per week. Participant scans were performed only when phantom measurements were within the predefined longitudinal QC range.

Blood samples were collected at baseline and at 1, 3, 6, and 12 months after treatment initiation to measure serum β-CTX, P1NP and PTH levels. All measurements were performed using electrochemiluminescence immunoassays on a Roche Cobas 6000 analyzer (e601 module; Roche Diagnostics, Mannheim, Germany). The intra-assay coefficients of variation (CVs) were <3% for β-CTX, <3% for PINP, and <2% for PTH, and the inter-assay CVs were <5% for β-CTX, <5% for PINP, and <4% for PTH, as specified by the manufacturer and confirmed by routine internal quality control. Health-related quality of life was evaluated using the Quality of Life Questionnaire of the European Foundation for Osteoporosis (ECOS-16) at baseline and at 6 and 12 months after treatment initiation. Fracture outcomes were collected during routine follow-up based on reported clinical events, and no protocol-specified blinded central adjudication or central image review was performed.

### 2.5. Adverse Events

All participants were questioned about AEs at each visit, and all were analyzed regardless of the investigators’ assessments of causality. The Medical Dictionary for Regulatory Activities (MedDRA) system was used to categorize reported AEs.

### 2.6. Statistical Analysis

This study had a power of 80% (with a two-sided α of 0.05) to detect a 1.8% ± 3.0% (mean ± standard deviation [SD]) difference in percent change in LS-BMD from baseline to 12 months between both groups. Assuming a 10% dropout rate, we planned to recruit a total of 100 randomized participants (50 per group).

All prespecified efficacy analyses were performed on the full analysis set (FAS) using a modified intention-to-treat principle. The FAS included data from all randomized participants who received at least one dose of the study drug with at least one post-baseline LS-BMD assessment. Participants without any evaluable post-baseline LS-BMD data were excluded from the FAS. The per-protocol set (PPS), defined as participants who met the eligibility criteria, completed all scheduled visits, had good treatment compliance (80–120%), and had no major protocol deviations or prohibited concomitant treatments, was also analyzed for effectiveness as a reference. Safety analyses were performed for all randomized participants who received at least one dose of the study drug.

We summarized continuous and categorical variables using descriptive statistics. Continuous variables are presented as means ± SDs or medians (interquartile ranges), and between-group comparisons were performed using the independent-samples *t*-test (two groups) or analysis of variance (multiple groups) for normally distributed variables; non-normally distributed variables were compared using non-parametric tests. Categorical variables were expressed as frequencies and percentages, with comparisons made using the Chi-square test or Fisher’s exact test when appropriate.

The primary analysis was performed using a linear mixed-effects model to evaluate between-group differences in BMD percent change from baseline to 12 months. Fixed-effect covariates included baseline BMD, baseline 25(OH)D stratification, visit time, treatment group, and the interaction between treatment group and visit time. The model accommodated missing values under the assumption of missing at random. An unstructured covariance matrix was used to model within-subject correlations.

To explore potential treatment differences and treatment-by-subgroup interactions, subgroup analyses were conducted according to age (≤65 vs. >65 years) and baseline serum 25(OH)D level (<20 vs. ≥20 ng/mL).

For the primary endpoint, missing values were imputed using the last-observation-carried-forward (LOCF) method. An analysis of covariance (ANCOVA) model was then applied, with treatment group and visit time used as categorical covariates, baseline BMD as a continuous covariate, and the interaction between treatment group and visit time included. ANCOVA with LOCF imputation was conducted as a sensitivity analysis. Additionally, a linear mixed-effects model was applied to the PPS to assess the consistency of results between the FAS and PPS populations.

All statistical tests were two-sided, and a *p* value < 0.05 was considered statistically significant. Statistical analyses were performed using R software, version 4.5.0 (R Foundation for Statistical Computing, Vienna, Austria).

## 3. Results

### 3.1. Participant Characteristics

**Figure 1:**
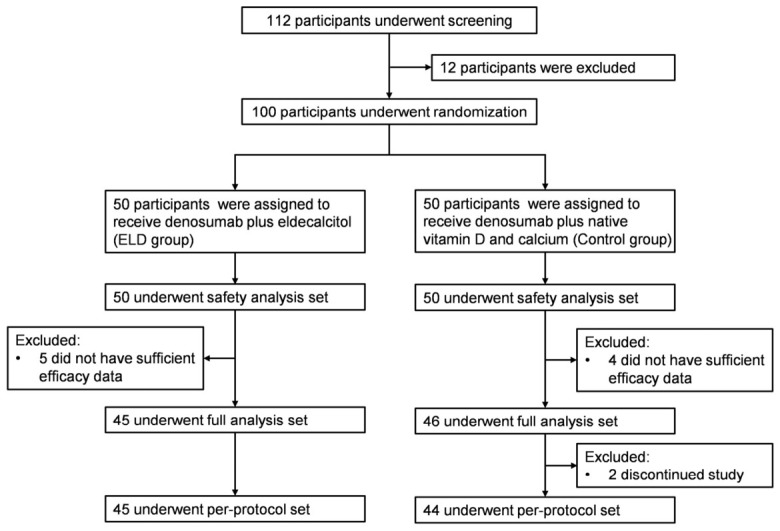
Flowchart depicting the steps involved in the recruitment process.

A total of 100 participants were enrolled in this study at our center in China and followed up for 12 months. Nine participants were excluded from the FAS because they had no evaluable post-baseline LS-BMD assessments available during the treatment period. The remaining 91 participants were included in the FAS (45 participants in the ELD group and 46 participants in the control group) (Figure 1). The baseline characteristics were balanced effectively between the two treatment groups (Table 1). The mean ages were 59.98 years in the ELD group and 60.57 years in the control group. Serum 25(OH)D levels at baseline in both groups were below 20 ng/mL.

### 3.2. BMD of Lumbar Spine, Total Hip and Femoral Neck

**Figure 2:**
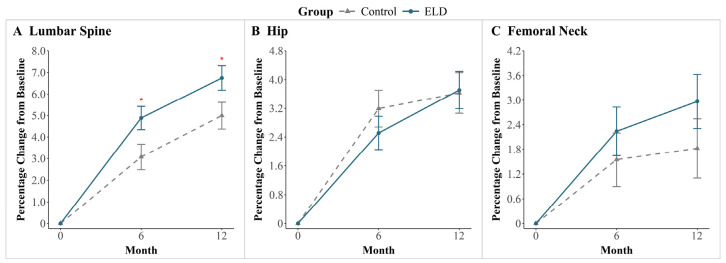
Percent change in bone mineral density from baseline at the lumbar spine (**A**), total hip (**B**), and femoral neck (**C**). * *p* < 0.05 versus the control group.

At baseline, LS-BMD (L1-L4) was 0.68 g/cm^2^ in the ELD group and 0.70 g/cm^2^ in the control group. At 12 months, these values increased to 0.73 and 0.74 g/cm^2^, respectively (Supplementary Table S1). The LS-BMD (L1–4) increased significantly from baseline at 6 and 12 months in both groups, with statistically greater least-squares mean percentage increases observed in the ELD group than in the control group at both time points (both *p* < 0.05). After 12 months of treatment, when used as the primary endpoint, the percent change in LS-BMD from baseline was 6.75% in the ELD group and 4.99% in the control group, yielding a significant between-group least-squares mean difference of 1.75% (95% confidence interval [CI], 0.10 to 3.41; *p* = 0.038) (Figure 2A). Sensitivity analyses using LOCF imputation followed by ANCOVA, as well as an additional model adjusting for baseline β-CTX, yielded results consistent with the primary analysis (Supplementary Table S2 for LOCF/ANCOVA; Supplementary Table S3 for β-CTX-adjusted analysis). Both groups showed significant improvements in TH-BMD and FN-BMD from baseline to 6 and 12 months (both *p* < 0.05), with no significant differences between groups (Figure 2B,C).

**Figure 3:**
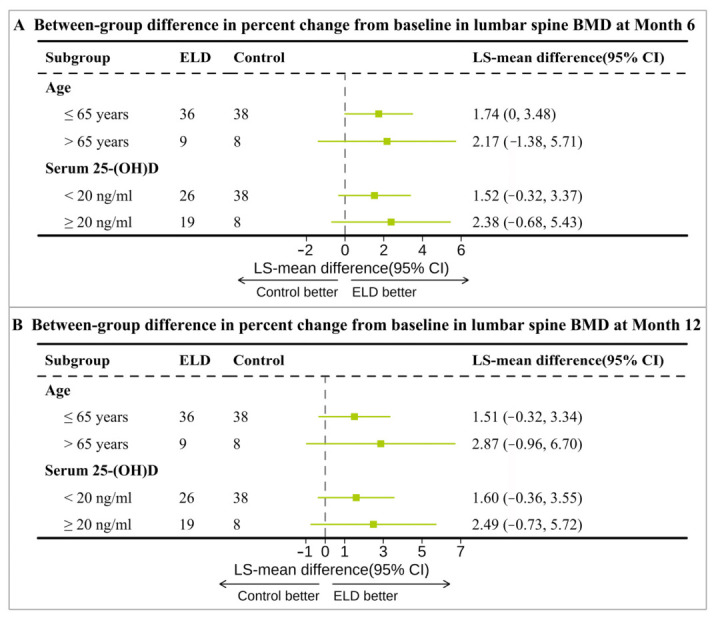
Forest plot of changes in bone mineral density from baseline across different populations.

Exploratory subgroup analyses of the effects of denosumab—combined with either ELD or native vitamin D plus calcium—on percent changes in LS-BMD at 6 and 12 months are shown in Figure 3. Across the age (≤65 vs. >65 years) and baseline serum 25(OH)D (<20 vs. ≥20 ng/mL) subgroups, the percent change in LS-BMD was consistently numerically higher in the ELD group than in the control group at both time points, although the 95% CIs were wide and crossed zero. No statistically significant treatment-by-subgroup interactions were observed.

### 3.3. Bone Turnover Markers and Serum PTH

**Figure 4:**
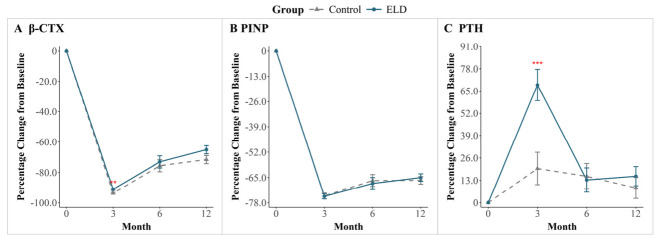
Percent change from baseline in bone turnover markers (β-CTX and PINP) and serum PTH. ** *p* < 0.01 versus the control group; *** *p* < 0.001 versus the control group.

Both β-CTX and PINP were markedly suppressed in both groups. At 3 months, serum β-CTX levels decreased by 91.00% from baseline in the ELD group versus 93.62% in the control group (least-squares mean difference in percent change, 2.62%; 95% CI, 0.98% to 4.27%; *p* = 0.002). This between-group difference was no longer evident at 6 months and was not statistically significant at 12 months (*p* = 0.092) (Figure 4A). Serum PINP levels showed no significant between-group differences at any time point (Figure 4B). Serum PTH levels showed a transient increase in the ELD group compared with the control group at 3 months (least-squares mean difference in percent change, 48.65%; 95% CI, 22.87% to 74.44%; *p* < 0.001), whereas no significant differences were observed at 6 or 12 months (Figure 4C). Between-group least-squares mean differences in β-CTX, PINP, and PTH are summarized in Supplementary Table S4.

### 3.4. Fractures and Quality of Life

During the 12-month treatment period, no new vertebral fractures occurred in the ELD group, whereas one new vertebral fracture occurred in the control group (1/46, 2.17%). Non-vertebral fractures occurred in one participant (1/45, 2.22%) in the ELD group, involving right humerus greater tuberosity, and in five participants (5/46, 10.87%) in the control group, involving the ulna, distal radius, hip, calcaneal, and lateral malleolus (Table 2). No statistical comparisons were performed for fracture outcomes because of the limited sample size and the relatively short duration of follow-up for evaluating fracture prevention.

At 6 and 12 months of treatment, no statistically significant differences in quality-of-life improvements were observed in between the ELD group and the control group (both *p* ≥ 0.05; Supplementary Table S5).

### 3.5. Adverse Events

During the study period, the total incidence of AEs was 54.0% (27/50, 35 events) in the ELD group and 48.0% (24/50, 36 events) in the control group, as listed in Table 3. The overall incidence of AEs did not differ between the two groups. The most common AE was secondary hyperparathyroidism, which occurred in 22 participants in the ELD group and 15 participants in the control group. Calcium-related AEs were rare, with one case of hypercalcemia in the ELD group and one case of increased blood calcium in the control group, and none were classified as serious. One participant in the ELD group discontinued treatment due to arrhythmia. One serious AE was reported in the control group and was not related to treatment. Overall, both treatment regimens were generally tolerated well, and no clinically significant safety concerns related to serum calcium abnormalities were observed in either group.

## 4. Discussion

This randomized clinical trial demonstrated that, in Chinese postmenopausal women with osteoporosis, denosumab combined with ELD resulted in a significantly greater improvement in LS-BMD at 12 months compared with denosumab combined with vitamin D and calcium supplementation, without increasing safety risks. These findings support the potential role of ELD as an adjunct to denosumab therapy in this population.

Previous studies have reported that supplementation with vitamin D and calcium during denosumab therapy helps maintain calcium homeostasis and enhance BMD gains [11,18], whereas active vitamin D may increase BMD more effectively than native vitamin D [16,17,19]. In the present study, both denosumab-based regimens led to significant increases in LS-, TH-, and FN-BMD from baseline. These findings align with previously reported Japanese data [17] and further support the greater effectiveness of ELD compared with native vitamin D plus calcium for increasing BMD in Chinese postmenopausal women with osteoporosis. However, because the control group received native vitamin D plus calcium supplementation and the ELD group did not receive protocol-mandated calcium supplementation, the observed between-group differences may reflect the combined effects of vitamin D formulation and calcium exposure rather than the effect of ELD alone. This design reflects real-world clinical practice, in which calcium is commonly prescribed with native vitamin D during denosumab therapy, whereas routine calcium supplementation may not be necessary with ELD because it enhances intestinal calcium absorption, and concomitant calcium may increase the risk of hypercalcemia. Consistent with this concern, a recent prospective observational cohort study in Chinese women with postmenopausal osteoporosis reported a higher incidence of hypercalcemia in patients receiving ELD with calcium supplementation than in those without [20]. Accordingly, our findings are best interpreted as a comparison of two adjunctive clinical strategies during denosumab therapy rather than a direct comparison of ELD alone versus native vitamin D alone.

In osteoporosis therapeutics, BMD is a well-established predictor of fracture risk, and epidemiological evidence indicates that fracture risk doubles for every 1-standard-deviation (10–15%) decrease in BMD [21]. In the present study, although a 1.75% additional increase in LS-BMD may appear modest in absolute terms, it represents a relative 35% greater gain compared with the control group (6.75% vs. 4.99%). Nevertheless, this trial was not designed or powered to assess fracture outcomes, and the 12-month follow-up was relatively short for evaluating anti-fracture efficacy. Accordingly, the additional LS-BMD improvement observed in the ELD group should not be interpreted as evidence of proven fracture-risk reduction, but rather as supportive of a potential skeletal benefit that warrants confirmation in larger and longer-term studies. Notably, the skeletal sites where between-group differences have been observed when using ELD vary across populations. In a previous Japanese study, ELD improved FN-BMD more than native vitamin D, with no between-group differences at the lumbar spine or total hip. In contrast, our Chinese study showed greater improvements in LS-BMD with ELD than with native vitamin D plus calcium at all time points. A previous Chinese randomized, double-blind, active-comparator phase III trial also reported a pronounced effect of ELD on LS-BMD [22]. Together, these findings suggest that ELD can enhance denosumab’s skeletal benefits, although the magnitude and anatomical pattern of these effects may differ across populations.

Mechanistically, the greater improvement in LS-BMD during postmenopausal osteoporosis observed when using ELD may be attributable to several factors. First, as a vitamin D receptor agonist, ELD enhances intestinal calcium absorption, optimizing serum calcium levels and thereby ensuring adequate mineral availability for bone formation [23]. Second, ELD promotes osteogenesis through bone minimodeling without relying solely on osteoclast inhibition [24]. Third, the slower responsiveness of cortical bone may contribute to site-specific differences, as cortical bone remodeling tends to be reactivated more slowly than trabecular bone in response to antiresorptive therapies [25], requiring longer-term cumulative effects to detect significant between-group differences and highlighting the need for extended follow-up to evaluate potential benefits at these sites. Additionally, the increase in BMD was driven mainly by denosumab, whereas the contribution of ELD appeared to be limited, consistent with previous observations [12].

Notably, the suppression of serum β-CTX and PINP levels reached its maximum at 3 months in both groups, and long-term suppression was broadly comparable thereafter. However, reduced suppression of β-CTX was observed at 3 months in the ELD group despite greater gains in LS-BMD at 12 months, suggesting that early changes in circulating bone turnover markers may not directly predict later BMD responses during potent antiresorptive therapy. While denosumab strongly suppresses bone resorption in both groups, the additional skeletal benefit of ELD may be mediated through mechanisms that are not fully captured by early β-CTX suppression alone, including enhanced intestinal calcium absorption, greater mineral availability, and possible effects on bone minimodeling. In addition, the higher baseline β-CTX level in the ELD group may have influenced the magnitude of early percentage change. Taken together, these findings suggest a complex, time-dependent interaction between ELD and denosumab, in which an early phase of metabolic re-adaptation may contribute to short-term fluctuations in bone turnover markers. The absence of persistent between-group differences in β-CTX and the lack of significant differences in PINP further indicate that the biological basis of this finding remains uncertain. Because both groups exhibited comparable suppression of β-CTX and PINP at 12 months, the concomitant use of ELD appears to have a limited long-term influence on the dynamics of bone metabolism markers during denosumab therapy [12].

In addition, a transient increase in serum PTH was observed at 3 months in the ELD group. This finding is clinically plausible and may be explained by the known pharmacological effects of denosumab. Previous studies have consistently shown that denosumab, through potent inhibition of osteoclastic bone resorption, can reduce calcium efflux from bone and subsequently induce a compensatory increase in PTH as part of calcium–PTH homeostatic feedback, particularly in the early phase of treatment [26]. This denosumab-associated PTH elevation has been reported across different populations and treatment settings [8,10]. In the present study, the early rise in PTH was more pronounced in the ELD group, which may reflect an interaction between denosumab-induced suppression of bone resorption and vitamin D receptor activation by ELD, leading to a short-term recalibration of calcium–PTH regulation. However, alternative or complementary explanations should also be considered. First, unlike the control group, which received 600 mg/day of elemental calcium, the ELD group did not receive exogenous calcium supplementation. Taking into account the marked suppression of bone resorption induced by denosumab, the absence of concomitant calcium supplementation may have resulted in a relatively less stable calcium balance during the early treatment phase, thereby triggering a more pronounced compensatory PTH elevation. Second, the higher incidence of reported secondary hyperparathyroidism in the ELD group may partly reflect detection and reporting bias, as the higher absolute PTH levels at 3 months led to a greater proportion of participants crossing the laboratory threshold, even though the underlying pathophysiological significance of this finding may be limited. Third, active vitamin D analogs may influence parathyroid calcium-sensing receptor expression or sensitivity, potentially altering the set point for PTH secretion in response to subtle changes in serum calcium [27]. The normalization of PTH levels at months 6 and 12 suggests a short-term adaptive response in calcium–PTH homeostasis rather than sustained secondary hyperparathyroidism. Importantly, this transient PTH elevation was not associated with adverse effects on BMD gains or calcium-related safety outcomes. It should be emphasized that the observed PTH elevation differs fundamentally from the persistent and clinically consequential secondary hyperparathyroidism observed in chronic kidney disease–mineral and bone disorder. The phenomenon in this study was transient, mild, and self-limiting, without concurrent hypocalcemia, hypercalcemia, or impairment of BMD gains. Nevertheless, careful monitoring of serum calcium and PTH during the first 3 months of combined therapy may be prudent, particularly in patients without concurrent calcium supplementation.

In terms of health-related quality of life, no significant differences were observed in ECOS-16 scores, suggesting that improvements in BMD may not immediately correspond to subjective symptomatic benefits, or that a longer duration of follow-up may be required to capture such changes.

With regard to safety, both denosumab-based regimens were generally well-tolerated, and no clinically significant safety concerns related to serum calcium abnormalities were observed in either group, consistent with previous data indicating that denosumab-induced hypocalcemia is uncommon in patients with preserved renal function who receive adequate calcium and vitamin D supplementation [28,29]. Nevertheless, given the potential risk of hypercalcemia associated with active vitamin D [30,31,32], careful use and enhanced monitoring remain essential, particularly in patients with impaired renal function. The overall incidence of AEs was comparable between groups throughout the study, supporting the safe use of ELD as a concomitant therapy during denosumab treatment.

This study has several limitations. First, the single-center design and relatively small sample size limited its power to detect differences in secondary endpoints, especially fractures and subgroup analyses. In addition, because calcium supplementation was only protocol-mandated in the control group and dietary calcium intake was not systematically collected, residual confounding related to total calcium exposure cannot be excluded. Second, the open-label design may introduce bias in patient-reported outcomes. Because no blinded central adjudication of fracture outcomes was prespecified, assessment bias cannot be completely excluded, although DXA scans were analyzed in a blinded manner and BMD and biochemical markers were objectively measured. Third, the 12-month follow-up precludes assessment of long-term fracture prevention and rare AEs. Finally, the study included only participants with normal renal function and Han Chinese ethnicity, which may limit generalizability to patients with renal impairment and to other ethnic groups, given documented differences in bone composition, BMD, skeletal geometry, and fracture risk across populations. These limitations highlight the need for multicenter, larger-scale, and longer-term randomized trials to confirm the efficacy and safety of denosumab combined with ELD, particularly regarding fracture risk, long-term safety, and use in patients with varying renal function and applicability across diverse populations.

## 5. Conclusions

In conclusion, this study is the first in China to compare the efficacy and safety of denosumab combined with either native vitamin D or ELD in postmenopausal women with osteoporosis. Denosumab combined with ELD improved LS-BMD more effectively than denosumab combined with native vitamin D plus calcium, suggesting that the addition of ELD may enhance the skeletal benefits of denosumab in postmenopausal osteoporosis. Larger multicenter studies involving more diverse populations should be conducted to confirm the reproducibility, external validity, and broader clinical applicability of these findings.

## Figures and Tables

**Table 1:** Participant characteristics prior to denosumab treatment.

Characteristic	ELD Group (*n* = 45)	Control Group (*n* = 46)
Age (years)	59.98 ± 6.11	60.57 ± 5.87
BMI (kg/m^2^)	23.06 ± 3.15	21.86 ± 2.80
Serum Alb (g/L)	46.69 ± 2.14	46.66 ± 1.87
Cre (μmol/L)	57.61 ± 9.29	57.03 ± 8.87
eGFR (mL/min)	94.63 ± 9.77	95.02 ± 9.21
Serum Ca (mmol/L)	2.39 ± 0.07	2.37 ± 0.07
Serum phosphorus (mmol/L)	1.24 ± 0.13	1.23 ± 0.15
Serum 25(OH)D (ng/mL)	19.54 ± 8.39	16.23 ± 4.21
Urinary Ca (mmol/L)	3.59 ± 2.66	3.45 ± 2.57
β-CTX (ng/mL)	0.69 ± 0.32 *	0.57 ± 0.19
PINP (ng/mL)	66.48 ± 21.83	61.90 ± 19.46
PTH (pg/mL)	42.97 ± 13.29	41.69 ± 9.98
BMD (g/cm^2^)		
LS (L1–4)	0.68 ± 0.06	0.70 ± 0.05
TH	0.72 ± 0.09	0.72 ± 0.09
FN	0.57 ± 0.07	0.59 ± 0.07
BMD T-score		
LS (L1-4)	−3.32 ± 0.57	−3.12 ± 0.48
TH	−1.84 ± 0.75	−1.83 ± 0.70
FN	−2.47 ± 0.64	−2.38 ± 0.67
History of prevalent vertebral fractures		
No	44	46
Yes	1	0
ECOS-16	1.94 ± 0.71	1.74 ± 0.50

Note: Data are means ± SDs or numbers. * *p* < 0.05 versus the control group. ELD, eldecalcitol; SD, standard deviation; BMI, body mass index; Alb, albumin; Cre, creatinine; eGFR, estimated glomerular filtration rate; Ca, calcium; β-CTX, β isomerized C-terminal telopeptide of type I collagen; PINP, procollagen type I N-terminal propeptide; PTH, parathyroid hormone; BMD, bone mineral density; LS, lumbar spine; TH, total hip; FN, femoral neck; ECOS-16, Quality of Life Questionnaire of the European Foundation for Osteoporosis.

**Table 2:** The incidence of new fractures at 12 months of treatment.

	ELD Group (*n* = 45)	Control Group (*n* = 46)
Fractures		
Left ulna fracture	0	1
Left distal radius fracture	0	1
Hip fracture	0	1
Right humerus greater tuberosity fracture	1	0
Left calcaneal fracture	0	1 *
Left lateral malleolus fracture	0	1 *
Thoracic vertebral fracture	0	1
No. of participants (%)	1 (2.22)	5 (10.87)

Note: ELD, eldecalcitol. * Multiple (≥2) fractures occurred in the same participant.

**Table 3:** Incidence of adverse events (safety analysis set).

Event	ELD Group (*n* = 50)	Control Group (*n* = 50)
Any adverse events during treatment period—no. (%)	27 (54.0)	24 (48.0)
Any serious adverse events—no. (%)	0 (0.0)	1 (2.0)
Adverse events leading to discontinuation—no. (%)		
Any event	1 (2.0)	0 (0.0)
Arrhythmia	1 (2.0)	0 (0.0)
Adverse events—no. (%)		
Secondary hyperparathyroidism	22 (44.0)	15 (30.0)
Cough	0 (0.0)	2 (4.0)
Dyspepsia	0 (0.0)	1 (2.0)
Upper respiratory tract infection	0 (0.0)	1 (2.0)
Constipation	0 (0.0)	1 (2.0)
Arthritis	1 (2.0)	1 (2.0)
Respiratory tract infection	0 (0.0)	1 (2.0)
Conjunctival hyperemia	0 (0.0)	1 (2.0)
Rotator cuff injury	0 (0.0)	1 (2.0)
Wrist joint stiffness	0 (0.0)	1 (2.0)
Knee osteoarthritis	0 (0.0)	1 (2.0)
Knee pain	0 (0.0)	1 (2.0)
Dermatitis	0 (0.0)	1 (2.0)
Chest pain	0 (0.0)	1 (2.0)
Ligament laxity	0 (0.0)	1 (2.0)
Knee joint disorder	0 (0.0)	1 (2.0)
Degenerative spondylitis	0 (0.0)	1 (2.0)
Nasopharyngeal ulcer	0 (0.0)	1 (2.0)
Arthralgia	1 (2.0)	0 (0.0)
Headache	1 (2.0)	0 (0.0)
Arrhythmia	1 (2.0)	0 (0.0)
Viral warts	1 (2.0)	0 (0.0)
Pain	1 (2.0)	0 (0.0)
Gastric pain	1 (2.0)	0 (0.0)
Intervertebral disc protrusion	1 (2.0)	0 (0.0)
Myofasciitis	1 (2.0)	0 (0.0)
Osteoarthritis	1 (2.0)	0 (0.0)
Hypercalcemia	1 (2.0)	0 (0.0)
Blood calcium increased	0 (0.0)	1 (2.0)

Note: ELD, eldecalcitol.

## Data Availability

The datasets used and/or analyzed during the current study are available from the corresponding author on reasonable request.

## Supplementary Materials

The following supporting information can be downloaded at https://www.mdpi.com/article/10.3390/jcm15145570/s1. Table S1: Bone mineral density at baseline, 6 and 12 months at the lumbar spine, total hip, and femoral neck; Table S2: Comparison of percent change from baseline in lumbar spine (L1–L4) BMD between the ELD group and the control group based on LOCF imputation and ANCOVA; Table S3: Comparison of percent change from baseline in lumbar spine (L1–L4) BMD between the ELD group and the control group based on β-CTX-adjusted analysis; Table S4: Least-squares mean differences in β-CTX, PINP, and PTH; Table S5: The differences in ECOS scores between the ELD group and the control group.

## References

[1] Ye C., Ebeling P., Kline G. (2025). Osteoporosis. Lancet.

[2] Morin S.N., Leslie W.D., Schousboe J.T. (2025). Osteoporosis: A review. JAMA.

[3] Walker M.D., Shane E. (2023). Postmenopausal osteoporosis. N. Engl. J. Med..

[4] Reid I.R., Bolland M.J. (2026). Management of postmenopausal osteoporosis. Endocr. Rev..

[5] Zeng Q., Li N., Wang Q., Feng J., Sun D., Zhang Q., Huang J., Wen Q., Hu R., Wang L. (2019). The prevalence of osteoporosis in China, a nationwide, multicenter DXA survey. J. Bone Miner. Res..

[6] Johnell O., Kanis J.A. (2006). An estimate of the worldwide prevalence and disability associated with osteoporotic fractures. Osteoporos. Int..

[7] Kendler D.L., Cosman F., Stad R.K., Ferrari S. (2022). Denosumab in the treatment of osteoporosis: 10 years later: A narrative review. Adv. Ther..

[8] Cummings S.R., San Martin J., McClung M.R., Siris E.S., Eastell R., Reid I.R., Delmas P., Zoog H.B., Austin M., Wang A. (2009). Denosumab for prevention of fractures in postmenopausal women with osteoporosis. N. Engl. J. Med..

[9] Curtis J.R., Arora T., Liu Y., Lin T.C., Spangler L., Brunetti V.C., Stad R.K., McDermott M., Bradbury B.D., Kim M. (2024). Comparative effectiveness of denosumab vs. alendronate among postmenopausal women with osteoporosis. J. Bone Miner. Res..

[10] Bone H.G., Wagman R.B., Brandi M.L., Brown J.P., Chapurlat R., Cummings S.R., Czerwiński E., Fahrleitner-Pammer A., Kendler D.L., Lippuner K. (2017). 10 years of denosumab treatment in postmenopausal women with osteoporosis: Results from the phase 3 randomised FREEDOM trial and open-label extension. Lancet Diabetes Endocrinol..

[11] Nakamura Y., Suzuki T., Kamimura M., Murakami K., Ikegami S., Uchiyama S., Kato H. (2017). Vitamin D and calcium are required at the time of denosumab administration during osteoporosis treatment. Bone Res..

[12] Saito S., Sugo Y., Tsuburai T., Kurasawa K., Nakamura T., Yoshikata H., Miyagi E., Sakakibara H. (2019). Activated vitamin D3 formulations can be safely used as concomitant medication for prevention of denosumab-induced hypocalcemia in women with postmenopausal osteoporosis. J. Obstet. Gynaecol. Res..

[13] Cambria M.T., Campanella M., Russo C., Agafonova A., Surdo S., Valle M.S., Malaguarnera L. (2026). Mechanistic actions of eldecalcitol and alfacalcidol across the muscle-metabolic axis: A selectivity perspective in sarcopenia and type 2 diabetes. iScience.

[14] Matsumoto T., Ito M., Hayashi Y., Hirota T., Tanigawara Y., Sone T., Fukunaga M., Shiraki M., Nakamura T. (2011). A new active vitamin D3 analog, eldecalcitol, prevents the risk of osteoporotic fractures—A randomized, active comparator, double-blind study. Bone.

[15] Uenishi K., Tokiwa M., Kato S., Shiraki M. (2018). Stimulation of intestinal calcium absorption by orally administrated vitamin D3 compounds: A prospective open-label randomized trial in osteoporosis. Osteoporos. Int..

[16] Ebina K., Kashii M., Hirao M., Hashimoto J., Noguchi T., Koizumi K., Kitaguchi K., Matsuoka H., Iwahashi T., Tsukamoto Y. (2017). Comparison of the effects of denosumab between a native vitamin D combination and an active vitamin D combination in patients with postmenopausal osteoporosis. J. Bone Miner. Metab..

[17] Suzuki T., Nakamura Y., Tanaka M., Kamimura M., Ikegami S., Uchiyama S., Kato H. (2018). Comparison of the effects of denosumab with either active vitamin D or native vitamin D on bone mineral density and bone turnover markers in postmenopausal osteoporosis. Mod. Rheumatol..

[18] Suzuki T., Nakamura Y., Kato H. (2018). Vitamin D and calcium addition during denosumab therapy over a period of four years significantly improves lumbar bone mineral density in Japanese osteoporosis patients. Nutrients.

[19] Yakabe M., Hosoi T., Matsumoto S., Fujimori K., Tamaki J., Nakatoh S., Ishii S., Okimoto N., Kamiya K., Akishita M. (2023). Prescription of vitamin D was associated with a lower incidence of hip fractures. Sci. Rep..

[20] Jiang Y., Zeng Y., Xin L., Chen W., Xu Y., Tian S., Wang Q., Li J., Chen S., Lin X. (2026). Eldecalcitol and calcium supplementation in Chinese women with postmenopausal osteoporosis: A prospective observational cohort study. J. Bone Miner. Metab..

[21] Divittorio G., Jackson K.L., Chindalore V.L., Welker W., Walker J.B. (2006). Examining the relationship between bone mineral density and fracture risk reduction during pharmacologic treatment of osteoporosis. Pharmacotherapy.

[22] Jiang Y., Tang H., Ma X., Cheng Q., Lin H., Jin X., Zhang Z., Yu W., He S., Kobayashi T. (2019). Eldecalcitol increases bone mineral density in Chinese osteoporotic patients without vitamin D or calcium supplementation. J. Bone Miner. Metab..

[23] Noguchi Y., Kawate H., Nomura M., Takayanagi R. (2013). Eldecalcitol for the treatment of osteoporosis. Clin. Interv. Aging.

[24] de Freitas P.H., Hasegawa T., Takeda S., Sasaki M., Tabata C., Oda K., Li M., Saito H., Amizuka N. (2011). Eldecalcitol, a second-generation vitamin D analog, drives bone minimodeling and reduces osteoclastic number in trabecular bone of ovariectomized rats. Bone.

[25] Langdahl B.L. (2021). Overview of treatment approaches to osteoporosis. Br. J. Pharmacol..

[26] Dempster D.W., Zhou H., Recker R.R., Brown J.P., Recknor C.P., Lewiecki E.M., Miller P.D., Rao S.D., Kendler D.L., Lindsay R. (2016). Differential effects of teriparatide and denosumab on intact PTH and bone formation indices: AVA osteoporosis study. J. Clin. Endocrinol. Metab..

[27] Hendy G.N., Canaff L. (2016). Calcium-Sensing Receptor Gene: Regulation of Expression. Front. Physiol..

[28] Cowan A., Jeyakumar N., McArthur E., Fleet J.L., Kanagalingam T., Karp I., Khan T., Muanda F.T., Nash D.M., Silver S.A. (2023). Hypocalcemia risk of denosumab across the spectrum of kidney disease: A population-based cohort study. J. Bone Miner. Res..

[29] Broadwell A., Chines A., Ebeling P.R., Franek E., Huang S., Smith S., Kendler D., Messina O., Miller P.D. (2021). Denosumab safety and efficacy among participants in the FREEDOM extension study with mild to moderate chronic kidney disease. J. Clin. Endocrinol. Metab..

[30] Aihara S., Yamada S., Oka H., Kamimura T., Nakano T., Tsuruya K., Harada A. (2019). Hypercalcemia and acute kidney injury induced by eldecalcitol in patients with osteoporosis: A case series of 32 patients at a single facility. Ren. Fail..

[31] Takeuchi Y., Saito H., Makishima M., Yokoyama H., Yamaguchi T., Fujii H., Inoue E., Isemura T., Kondo S. (2022). Long-term safety of eldecalcitol in Japanese patients with osteoporosis: A retrospective, large-scale database study. J. Bone Miner. Metab..

[32] Ri K., Fukasawa T., Masuda S., Tanaka S., Takeuchi M., Yoshida S., Kawakami K. (2023). Frequency and determinants of serum calcium monitoring during eldecalcitol therapy in patients with osteoporosis. J. Bone Miner. Metab..

