# Supplementary material for: Comparison of Efficacy and Safety of Denosumab with Eldecalcitol or Native Vitamin D in Postmenopausal Chinese Women with Osteoporosis (ESCORT): A Randomized Controlled Trial
Source: J Clin Med. 2026 Jul 16;15(14):5570. doi: 10.3390/jcm15145570 (PMC13412344; doi:10.3390/jcm15145570)
Supplement: Supplementary file 1 [file jcm-15-05570-s001.zip › jcm-4369630-supplementary.pdf]

Table S1. Bone mineral density at baseline, 6 and 12 months at the lumbar spine, total hip, and femoral neck

BMD (g/cm²)	Follow-up	ELD group	Control group	*P* value
LS (L1–4)	Baseline	0.68 ± 0.06	0.70 ± 0.05	0.081
	6 M	0.71 ± 0.07	0.72 ± 0.06	0.450
	12 M	0.73 ± 0.07	0.74 ± 0.06	0.466
TH	Baseline	0.72 ± 0.09	0.72 ± 0.09	0.962
	6 M	0.73 ± 0.08	0.74 ± 0.09	0.631
	12 M	0.74 ± 0.08	0.75 ± 0.09	0.876
FN	Baseline	0.57 ± 0.07	0.59 ± 0.07	0.248
	6 M	0.59 ± 0.07	0.60 ± 0.07	0.354
	12 M	0.59 ± 0.07	0.60 ± 0.08	0.574

Note: Data are means ± SD or number.

BMD, bone mineral density; LS, lumbar spine; TH, total hip; FN, femoral neck; ELD, eldecalcitol.

Table S2. Comparison of percent change from baseline in lumbar spine (L1–L4) BMD between the ELD group and the control group based on LOCF imputation and ANCOVA.

Follow-up	ELD group (95% CI)	Control group (95% CI)	Least-squares mean change (95% CI)	*P* value
Baseline	-0.13 (-1.02, 0.76)	-0.07 (-0.99, 0.86)	-0.06 (-1.34, 1.21)	0.923
6 M	4.95 (4.05, 5.84)	3.01 (2.09, 3.93)	1.94 (0.66, 3.21)	0.003
12 M	6.81 (5.92, 7.70)	4.95 (4.03, 5.87)	1.86 (0.58, 3.14)	0.004

Note: BMD, bone mineral density; ELD, eldecalcitol; LOCF, last observation carried forward; ANCOVA, analysis of covariance; CI, confidence interval.

Table S3. Comparison of percent change from baseline in lumbar spine (L1–L4) BMD between the ELD group and the control group based on β-CTX-adjusted analysis

Follow-up	ELD group (95% CI)	Control group (95% CI)	Least-squares mean change (95% CI)	*P* value
6 M	5.13 (3.68, 6.59)	3.39 (1.67, 5.11)	1.74 (0.14, 3.34)	0.0333
12 M	7.00 (5.50, 8.50)	5.31 (3.55, 7.07)	1.69 (0, 3.37)	0.0496

Note: BMD, bone mineral density; ELD, eldecalcitol; β-CTX, β isomerized C-terminal telopeptide of type I collagen; CI, confidence interval.

Table S4. Least-squares mean differences in β-CTX, PINP, and PTH.

	Follow-up	ELD group (95% CI)	Control group (95% CI)	Least-squares mean change (95% CI)	*P* value
β-CTX	Baseline	0.69 (0.61, 0.77)	0.57 (0.50, 0.65)	0.11 (0, 0.23)	0.042
	3 M	0.05 (0.04, 0.05)	0.03 (0.03, 0.04)	0.01 (0, 0.02)	0.002
	6 M	0.16 (0.12, 0.20)	0.13 (0.09, 0.17)	0.03 (-0.03, 0.09)	0.256
	12 M	0.21 (0.17, 0.25)	0.16 (0.12, 0.20)	0.05 (0, 0.11)	0.057
PINP	Baseline	66.61 (60.48, 72.73)	62.43 (56.33, 68.54)	4.17 (-4.46, 12.80)	0.339
	3 M	15.27 (13.61, 16.92)	15.22 (13.38, 17.05)	0.05 (-2.36, 2.46)	0.968
	6 M	19.67 (14.91, 24.43)	20.52 (15.74, 25.30)	-0.84 (-7.57, 5.88)	0.803
	12 M	21.33 (19.08, 23.59)	19.45 (17.06, 21.83)	1.89 (-1.34, 5.12)	0.249
PTH	Baseline	42.76 (39.27, 46.24)	40.80 (37.01, 44.59)	1.95 (-3.07, 6.98)	0.442
	3 M	72.18 (61.96, 82.39)	47.84 (37.53, 58.15)	24.34 (9.87, 38.81)	0.001
	6 M	47.03 (40.53, 53.54)	45.48 (38.86, 52.11)	1.55 (-7.67, 10.76)	0.739
	12 M	47.15 (42.60, 51.70)	42.10 (37.31, 46.89)	5.05 (-1.45, 11.55)	0.126

Note: β-CTX, β isomerized C-terminal telopeptide of type I collagen; PINP, procollagen type I N-terminal propeptide; PTH, parathyroid hormone; ELD, eldecalcitol; CI, confidence interval.

Table S5. The differences in ECOS scores between the ELD group and the control group.

Follow-up	ELD group (95% CI)	Control group (95% CI)	Least-squares mean change (95% CI)	*P* value
6 M	-0.12 (-0.24, 0.01)	-0.21 (-0.34, -0.08)	0.09 (-0.09, 0.27)	0.309
12 M	-0.05 (-0.17, 0.08)	-0.02 (-0.16, 0.11)	-0.02 (-0.21, 0.16)	0.823

Note: ECOS, Quality of Life Questionnaire of the European Foundation for Osteoporosis; ELD, eldecalcitol; CI, confidence interval.
